# Technical reporting in digital forensics

**DOI:** 10.1111/1556-4029.15108

**Published:** 2022-08-15

**Authors:** Graeme Horsman

**Affiliations:** ^1^ Cranfield University Bedford UK

**Keywords:** digital evidence, digital forensics, report writing, technical reporting

## Abstract

One of the primary roles of a practitioner in the field of digital forensics (DF) is to conduct the examination of any lawfully seized digital device content and report upon any findings that may support an inquiry being conducted. While there are many intricacies to this task, in some cases, an inquiry will commence with a practitioner carrying out the necessary examination work required to report any findings at a “technical level.” Such technical reports are often used for intelligence gathering purposes in an attempt to establish the potential evidential value of a device or data set and are often a precursor to, and catalyst for, further and often more extensive forensic work being commissioned. Therefore, the ability to report at a technical level should be considered a fundamental skill required of all practitioners in this discipline and any attempts to provide guidance and support for conducting this task effectively should be encouraged. This work explores the role of technical reporting, where a series of reporting examples are presented that explore the intricacies involved with conveying digital forensic findings at a technical level. Procedural and linguistic challenges are investigated and evaluated in order to acknowledge the pitfalls that practitioners may encounter and to identify potential technical reporting best practices.

**Highlights**

- The importance of reporting is outlined in the context of digital forensic examinations.
- Reporting at a “technical” level is discussed and methods for doing this are proposed.
- The structure of technical reports is evaluated, and examples for their construction are offered and explained.

## 1 INTRODUCTION

Practitioners operating within a digital forensic (DF) laboratory are required to communicate the results of any examination they carry out to their clients appropriately and effectively [1, 2], a task that is often far from simple. This communication frequently takes the form of a report, which having followed any instructions provided from their client should outline those findings considered to be relevant to the inquiry being conducted. In an alleged incident where a digital device may help to ascertain what has occurred, a DF practitioner's report is likely to play an important role in regard to both any investigative team's immediate comprehension of the event and their future decision making. Prior to commencing an examination, the DF practitioner must identify the needs of their client and ensure these sit within the remit of their operational role to ensure any forensic work conducted will meet the needs of all those involved in the investigative process. Fundamentally, this includes the practitioner establishing how their client requires them to report upon any findings. The practitioner may be commissioned to report at a “technical level” or, providing they are capable, they may be required to provide an investigative or evaluative opinion in regard to any of their findings, having also conducted the necessary work needed to facilitate this. In defining the DF practitioner, reference to the College of Policing [31] is made which state that the role of the practitioner is to “support and advise on the delivery of digital services for major crimes, incidents, operations or any investigation that requires specialist DF investigative assistance, applying a broad range of DFs techniques to support the investigative strategy.” Those in this role require the training and skills to operate forensic tools to effectively examine digital devices and their content [31].

Any practitioner reporting investigatively or evaluatively does so with the expectation that their opinion with regard to any findings and their meaning is included where required and conveyed appropriately [3]. Investigative reporting governs the practitioner to express any opinions merely as “possibilities” or “explanations of observations,” where evaluative reporting requires the formation of any opinion to be based upon at least one paired proposition and any uncertainty regarding the opinion to be evaluated and explained [3]. In contrast, practitioners reporting at a technical level do so without providing any opinion in regard to the meaning of any data identified as part of the examination, instead, only stating relevant and objective technical information about it and the processes undertaken—in essence, a “technical description” [4]. While we may consider there to be these three types of “*reporting‐level*” in DF [2], practitioners in some jurisdictions may be required to report at a technical level more often than investigatively or evaluatively. Technical reporting is often a precursor to other report types in a case, and in some instances, it can be a determinative factor as to whether further forensic work is required as part of an investigation [3]. The role of a technical report is often to provide initial case intelligence, which may support later charging decisions and shape further forensic workflows, seen with initiatives such as Streamlined Forensic Reporting in England and Wales [5]. Therefore, the ability to effectively report at a technical level is a core skill that a DF practitioner must possess given its position within the typical DF investigative process, and its potential impact on any later stages of it.

While literature focusing on reporting best practices specific to DF is available [2], along with vendor and blog‐based commentaries [6, 7, 8, 9], fundamental contributions have also been made in the wider forensic science communities (see [3, 4, 10, 11]), where lessons can be learned. However, these works often focus on the communication of opinion evidence where commentary regarding best practices for technical reporting appear somewhat limited. As a result, this work explores the fundamental role that technical reporting plays as part of a DF examination, building upon previous discussions by Horsman [2], where a series of reporting examples are presented that explore the intricacies involved with conveying DF findings at a technical level. Procedural and linguistic challenges are investigated and evaluated in order to suggest both the pitfalls that practitioners may encounter and potential technical reporting best practices. It should be made clear that this work recognizes that different jurisdictions may operate differing reporting practices and therefore any suggestions made should be evaluated against a practitioner's legal/contractual requirements.

## 2 WHAT IS TECHNICAL REPORTING?

The concept of a technical report in DF may be misconstrued due to an assumed link between the term “technical” and “technology” within the context of reporting. Individuals may wonder why all reports produced by DF practitioners are not considered technical reports, given they are often the esult of an examination of some form of technology product. The term “technology” denotes the media investigated as part of any DF examination—the target focus of the report and forensic process; however, practitioners do not refer to their reports colloquially as “technology reports.” Instead, arguably the appropriate term is that of a “technical report,” which is considered a reporting product—a type of report produced by a practitioner that adheres to the rules of technical reporting and contains those characteristics required of a report that is conveying findings at a technical/factual level. There are several formal descriptions of technical reporting offered by the wider forensic science community, and reference to these is made here in order to support this discussion.

In 2009, the Association of Forensic Science Providers outline technical (also referred to as “factual”) reporting as the following:

This is the ‘factual’ reporting of a test outcome based solely on the technical competence of the individual. No inferences/explanations (opinion) are drawn from the test results (observations) — a ‘technical’ interpretation is provided’ [12].

In addition, The European Network of Forensic Science Institutes's (ENFSI) “guidelines for best practice in the forensic examination of digital technology” defines technical reporting as “the ‘factual’ reporting of a test outcome based solely on the technical competence of the individual. No inferences/explanations (opinion) are drawn from the test results (observations)” [13]. This stance is confirmed by the Forensic Science Regulator [4 at p.47–48] who states:

“A factual report explains what the practitioner has done and the observations obtained, such as a list of the files of a certain type that were retrieved. It offers no opinion on how the files came to be on the device or whether any of their content is relevant to a fact in issue in the case.”

Willis et al., [3 at p.26] state that “in a strict sense, purely technical or factual reporting amounts to a descriptive account of findings,” acknowledged also by Champod et al., [10], Biedermann et al., [14], Ballantyneet al., [15] and Sunde and Horsman [16]. Biedermann and Vuille, [17 at p.89] state that the technical report “amounts, in essence, to the reporting of observations in a descriptive way without elaborating on any propositions, except where propositions relate to general categories of classification.” In relation to the field of DF, Brown [18 at p.66] considers the technical reporting task as “producing highly granular reports detailing the inner workings of information communication technologies, file integrity, authenticity of the information, and movement of data” [18 at p.66]. The Forensic Science Regulators Codes of Practice and Conduct for DF services distinguish technical reports from reports that are evaluative in nature [29].

It is important to note that not all reporting involves the giving of an opinion in regard to a practitioner's interpretation of data. Some jurisdictions or law enforcement laboratories may operate procedures that require a DF practitioner to initially report at a technical level, where the Forensic Science Regulator [4 at p.48] acknowledges the role of many practitioners is as “a scientist or technician involved in the generation of observations and/or factual reports, but who is not qualified to give expert evidence.” This position is indicated by ENFSI [13] who states that “a forensic scientist/officer who has achieved levels of technical competency for specific equipment and services…are able to write reports and statements of factual information in their specific specialist areas and can provide factual testimony in court” [13 at p.6].

### 2.1 Technical reporting in practice

Engagement with factual/technical reporting in DF may differ jurisdictionally; however, its role in England and Wales is acknowledged by initiatives such as “Streamlined Forensic Reporting” [5]. Particularly in cases where investigative inquiries focus on the identification of indecent images of children, Streamlined Forensic Reporting can help to make more efficient charging decisions through the early production of a technical report that technically describes the presence of illegal content on a system [5]. Reporting at a technical level in most cases will be driven by the information and instructions provided by the client. In addition, a practitioner may not actually be qualified to report at anything other (i.e., opinion‐based) than a technical level. As in criminal cases, this client will often be the officer in charge of the investigation, where their understanding of the alleged offense and surrounding circumstances will guide the development of a set of digital examination actions to be undertaken, along with technical input from the practitioner.

Where a practitioner is commissioned to conduct a DF examination and produce a technical report, findings should be presented absent of interpretation, with only an objective technical description of them provided. Practitioners should be aware that in order to report upon the meaning of any data found, any assessment of meaning often requires additional investigative work to be conducted in order to test and evaluate any investigative hypotheses that they may possess for the purposes of reliability and quality assurance. If this work has not been conducted, then any findings should not be conveyed in any form beyond that of a technical level, even if that was requested by a client. In some cases, following the submission of a technical report to a client, additional investigatory requirements may be established that obligate the practitioner to conduct further work and report at a level that permits them to express their opinion in regard to the meaning of any relevant data, whether that be investigative or evaluative.

*To note: It is important to acknowledge that in some cases, reporting at a technical level will not be appropriate for meeting the needs of the client, particularly in complex cases where an interpretation of the meaning of any data goes beyond a simple technical description is required [5]. Where this is the case, an appropriate dialogue should take place with all parties so that the best and most appropriate course of investigatory action can be identified and agreed upon going forward.

### 2.2 Technical reporting content

Following existing guidance regarding technical/factual reporting, it is clear that the role it plays within DF is one that is reserved for providing an objective technical description of data found within an examination as a result of processes/procedures deployed by the practitioner and in line with instructions provided by their client. While this may appear straightforward, the confines that this reporting approach operates within require stating so as to ensure that a practitioner does not overstep its remit. Horsman [2] suggests that technical reports will report upon any processes used, and the quantity, location, and type of data found as a result of them. At a conceptual level, it is suggested that the contents of technical reports will predominantly focus on communicating findings using the following three constructs:

1. Reporting upon the use of a process/procedure (the tool used) and the configuration and remit of it with an examination.
2. Reporting the results presented by a process/procedure (the tool used).
3. Any data specifically highlighted by the practitioner, and the criteria used to do this.

To provide context to the use of the three above‐mentioned constructs, consider the following example of a technical report that comments on the presence of Internet history on a device. Any statement made within the report should describe the processes used to find and parse Internet history (1), before stating any presented results (2) and where necessary highlighting specific records (for example to known bad websites) using filtering criteria (3). Not all three categories are required for every action conducted by the practitioner.

The investigative work conducted for the purpose of technical reporting is unlikely to be as thorough in terms of data analysis than in cases where investigative or evaluative opinion and the interpretation or meaning are required. This is particularly the case when a technical report is commissioned for the purposes of intelligence gathering, where an aim may be to reach a reliable and quick decision with regards to the relevance of a particular exhibit to an inquiry and whether any further examination of it is required. Reporting at a technical level is in most cases likely to be predominantly driven by the use of automated processes/procedures that are used to search and sift data in order to establish the presence of content that may be useful to the overarching inquiry being conducted. For that reason, any results presented are often those provided by the process/procedure utilized within an examination, and this must be clear when reported.

All forensic reports must convey their contents accurately [25, 26], where the same is true for technical reports in DF; however, this is not the only reporting challenge faced by the practitioner. Practitioners must be aware of how they linguistically construct and communicate the findings via their report and understand the impact of any language used.

#### 2.2.1 Constructing the report

It is also important to note that while there is some guidance and published standards for technical reporting in DF, there will inevitably have been internal developments of reporting practices at organizations. This means it is likely that to some degree, divergent reporting approaches are possibly taken between DF organizations, at a time where consistency is being strived for in forensic practices as it is perceived as a mark of quality assurance [19, 20, 25]. Further, where bad reporting practice is in operation, such practices may be internally “recycled” without any evaluation of their quality, leading to their perpetual use.

When reporting at a technical level, practitioners must be aware of the language they are using to convey their findings and ensure that it does so in a manner that is appropriate for a technical report. Through loosely constructed statements, a practitioner may use phrasing that insinuates or presents an opinion with regard to the meaning of any findings, or use language which could misrepresent the impact or importance of any findings with regard to the case being examined [2]. This may be done intentionally where the practitioner does not understand the remit of a technical report and oversteps their duty, feeling obliged to comment on findings in a way that sits beyond the rules of technical reporting. Alternatively, the practitioner may misunderstand or fail to recognize the potential ways that the language they have used to convey findings could be interpreted.

The contents of a technical report must convey “procedural certainty” in regards to any findings and how/why they have been identified. In line with the Forensic Science Regulators [4 at p.47–48], the report must explain “what the practitioner has done and the observations obtained, such as a list of the files of a certain type that were retrieved,” placing emphasis on the need to be explicitly clear with regards to what procedure/process has been used and the configuration of it which has subsequently led to any presented results [26]. In many cases, it must be clear what tool is responsible for any presented results and how it has derived these—its settings and purpose.

These challenges in relation to the construction of technical reports are explored through a series of contextualized examples.

## 3 SOME WORKED EXAMPLES

In order to examine the challenges associated with constructing a technical report, this work will explore how relevant data can be conveyed through the provision of worked examples, building on initial comments and work regarding technical reporting provided by Horsman [2]. In order to maximize the value and transferability of the examples provided, a focus will be maintained on the main three constructs of technical reporting noted in Section 2.2 and restated here:

1. Reporting upon the use of a process/procedure (the tool used) and the configuration and remit of it.
2. Reporting the results presented by a process/procedure (the tool used).
3. Any data specifically highlighted by the practitioner, and the criteria used to do this.

Examples of common tasks conveyed in technical reports will be used to explore both effective approaches to reporting each of these three constructs, as well as highlighting potential pitfalls.

*To note:‐ *The structure and format of a technical report are not considered*, *this exercise focuses only on the presentation of findings at a technical level*. *The construction of sections such as the practitioner*'*s declaration of relevant experience and pre‐examination continuity and integrity checks and not explored*.

### 3.1 Reporting upon the use of a process/procedure (the tool used) and the configuration and remit of it

As part of any DF examination, practitioners will often engage appropriate and approved forensic processes/procedures in order to search, identify, parse, and display relevant content that may exist on a seized device. The result of these processes/procedures often forms a subset of information upon which a practitioner may report upon [24]. At a technical reporting level, findings are predominantly tool‐driven, i.e., the practitioner will run or conduct a process/procedure and simply report the output that is presented by the tool. It should be made clear that any results are only the product of the tool/process/procedure being used, as the purpose of technical reporting is not to interpret the meaning of any data. Following this point, it is important to refer back to the purpose of a technical report ‐ to report upon procedures conducted and any observed outcomes. For that reason, when a technical report describes the use of a process/procedure, regardless of what it is, it is suggested that the following information must be conveyed in any statement given by the practitioner:

1. The name of the process/procedure being used.
2. The name and version of the tool used to conduct the process/procedure.
3. A technical description of what the process/procedure is designed to do.
4. How the process has been configured for use in the current case, for example, process/procedure *P* has been used to recover files of type *X*, *Y*, and *Z using settings A & B*.
5. The completion status of the process/procedure, for example, whether it has fully completed, partially completed, any reported errors, etc.
6. The results provided by the tool carrying out the process/procedure.

It is suggested that taking into account the six criteria above, the following structure can be adopted when communicating the use of a process/procedure in a technical report.

I have carried out <*process/procedure name*> using <*tools name & version number*>. This process/procedure is used for <*description of process/procedure purpose*>. This process/procedure has been used to <*description of why this process/procedure has been used in the current case*>. To provide an overview, the process/procedure is configured to <*simple description of relevant configuration settings if required*>, however, full details of the process/procedure's configuration settings are available on request. The process/procedure completed <*describe completion status i.e., fully, partially*, *list of errors & impact of these on any results..*.>. The results presented by <*process name*> are found here: <*present/exhibit a list of results provided*>.

*Important: It is acknowledged that practitioners will not always supply all data resulting from a completed process/procedure where in some cases outputted results may be filtered using appropriate criteria which may be defined by the client or wider investigative team. This refers to the third of the three constructs of technical reporting noted above where a discussion on how to convey when a data set has been filtered by the practitioner is reserved for Section 3.3. Here, the focus will remain on how to describe the use of a process and its presented results in their totality.

The first example provided involves the consideration of the use of a deleted file recovery process.

#### 3.1.1 Deleted file recovery

Practitioners are often required to recover files of specific types from nonlive areas of a device's storage media, termed deleted file recovery [21]. Of course, such processes can be complex depending upon the configuration of them where practitioners should ensure that they understand the full remit of the process they intend to deploy prior to deploying it, to ensure that any description of it is correct. If we consider a hypothetical “simple” consideration of deleted file recovery processes/procedures, it is suggested that the use of deleted file recovery process/procedures may be conveyed with the following structured statement:

Deleted file recovery processes were conducted on <*exhibit name*> using <*tools name & version number*>. Deleted file recovery is a process that attempts to identify and recover files from a device that are no longer live and therefore accessible to the user without the use of specialist software. Full details of the configuration of the deleted file recovery process are available on request. A deleted file recovery process was conducted in an attempt to identify and recover files of types <*list of file types*>. This process completed <*describe completion status i.e. fully*, *partially*, *list of errors & impact of these on any results..*.>. <*tools name & version number*> reported that <*X amount*> files of type <*file type*> had been recovered.

The above statement intends to offer a structure, where practitioners may opt to include their own definition/description of “file deletion” and any associated terms. In turn, they may wish to further explain the remit of such a process if they are using it to also carve data from within compound files etc. as well as unallocated areas of any storage media. The above statement enforces the need to make reference to the tool used to conduct the recovery process and its version number, where it is assumed that in most instances file recovery will be via the use of a specialist tool. It is important to note that different tools may produce dissimilar (either in full, however more likely in part) results either due to a difference in capability, or performance issues may be present but undetected (errors). For this reason, stating a tool's name and version number allows scrutiny of the work conducted to ensure that tools have not been deployed that have known flaws, or where a tool is subsequently found later to have file recovery limitations, such work could be flagged as unsafe and revisited.

Where a practitioner is simply presenting the results provided by a process/procedure, the accuracy of these is only as good as the ability of the tool used and its configuration, making it important that sufficient information is provided to critically evaluate this undertaking. When discussing the process/procedure and particularly the configuration of it, it is important to balance the need for thoroughness and transparency with the possibility to cause technical distraction and confusion within the statement itself. Many processes/procedures can be configured in many different ways, where the full extent of their setup may impact the clarity of the presented findings if fully laid out within a technical statement. In addition, one of the fundamental practices of reporting in DF is to ensure the contents of a report is provided in a manner that is accessible to its target audience [2, 6].

For that reason, it may be more appropriate to explain the purpose of the process/procedure, why it has been deployed in the current case and to highlight any main relevant configurations within the report itself. However, practitioners should be prepared to supply full details of the configuration of their processes/procedures if required, where such details should be retrievable from the practitioner's contemporaneous notes [22, 30]. This approach should not be considered an opportunity to obfuscate any work undertaken, where practitioners must determine what amount of technical configuration information is appropriate in any given circumstances.

In contrast to the structure noted above, two alternative example statements are provided below which arguably could also be used to describe the file recovery process, derived from Horsman [2]. These statements are not derived from actual reports, they are merely envisaged examples.

1. I have found <*X*> number of files of type <*file type*>.
2. There are <*X*> number of files of type <*file type*> present on <*exhibit device*> which have been recovered.

Statement 1 although concise, fails to provide enough information to convey procedural certainty, i.e. *how have these files been found and where from*? The language “*I have found*” insufficiently conveys how the files were identified and what process/procedure was conducted, where it is likely that a tool has been used to find relevant content and any results provided by it have been described by the practitioner. The phrasing in statement 1 means it is difficult to establish whether any method used is appropriate and robust because details of the process are unknown. The language also creates ambiguity as the practitioner is unlikely to have found the files, rather their tool has. In most cases, the tool will have presented the practitioner with results, and they will have sought to report such findings, where it is improbable that every instance has been manually verified.

While statement 2 maintains many of the same issues as statement 1, it raises a separate concern stemming from the language “*there are <X> number*.” This is definitive language conveying a message that the number of files stated to be present is absolute. In reality, the process/procedure used may have limitations, be configured in a way that results in relevant files being missed, or, alternative tools may be more effective in identifying more data present on a device than the one being used. In essence, it is difficult to be absolutely certain of the amount of data that resides on a device as in many cases, particularly when conducting work that is appropriate to a technical reporting level, where any attempt to quantify this by the practitioner is likely to simply rely upon what their tool presents. This situation is also alluded to by the Forensic Science Regulator [4]. As a result, it should be clear that any reported quantity of deleted content is a quantification made by the tool used to conduct the process.

#### 3.1.2 Keyword searching

As a second example, the following structure is proposed for describing the use of a keyword/string search:

Utilising <*tools name & version number*>, keyword searches were conducted for the following criteria <*list of criteria*>. The keyword search criteria were provided by <*description of how the keyword search criteria were derived*>. The process of keyword searching involves the systematic processing of a target device's data in order to identify whether any of the keyword criteria searched for are present in it. Details of the technical configuration for the tool used to conduct these keyword searches are available on request. This process completed <*describe completion status i.e. fully, partially, list of errors & impact of these on any results..*.>. On completion of the process, <*tools name & version number*> recorded <*X*> number of matches to the keyword search criteria, referred to as ‘keyword hits’.

<*Presentation of keyword hit metadata*>

Keyword searching process/procedures, like those concerning deleted file recovery, can be complex in terms of configuration and deployment, therefore many of the points raised in Section 3.1.1 also apply here. Within the remit of a technical report, it is important that those keyword criteria searched for are listed and available for review so that it is possible to ascertain the extent of the search and any potential limitations of it. In terms of limitations and for the purposes of search transparency, it may be necessary to state those scenarios where a keyword search's ability may be limited—for example, data inside encrypted containers may not be searchable by standard keyword searching algorithms if the ability to decrypt it is not available. It is also suggested that any statement includes a description of how the keyword search criteria were derived, for example, whether they have been supplied by any officer in charge of the investigation or generated by the practitioner themselves

In addition, given the potentially large number of intricacies involved with the configuration of a keyword search, it is considered necessary to state that the details of the technical configuration for the tool used to conduct the keyword searches are available on request—imilar to the scenario noted with file recovery processes/procedures. However, for transparency and thoroughness, practitioners may consider supplying an “overview statement” describing those areas searched, and accompany this where necessary with a description of meaning—i.e. live files only, sector‐level searches etc.’ however, this may be considered optional and practitioners should exercise caution so as to not cause confusion. Any resulting hits should be listed and available for review.

#### 3.1.3 Parsing data

Practitioners will also utilize tools designed to parse and present data contained within specific files that they identify as part of their examination where examples may include tools for parsing Internet history data or chat logs. Where a data parsing process is deployed, the following statement structure is suggested for conveying the use of this type of process/procedure.

File <*F*> was present on <*exhibit device*> at <*location*>. <*F*> contains information about <*description of the purpose of F and its relevant technical content*>. Utilising <*tools name & version number*> the contents of <*F*> were parsed and findings are displayed here: <*list of findings*>. The process completed and reported <*details of completion status i.e. errors, no errors etc*>.

Perhaps the most important part of describing the use of a parse and present process is to ensure that a description of the purpose of any file being parsed and its content is provided. This ensures that the technical purpose of parsing any file using this process/procedure should be clear and understandable. Some parse and present processes/procedures may be conducted with standalone tools, where it is important to ensure the name and version number of the tool is supplied, and in some cases, it may be necessary to provide details of where the tool originated from. Any internally developed parse and present processes/procedures should be fully documented and a practitioner should be prepared to disclose any validation/verification information which demonstrates the reliability of it if required.

#### 3.1.4 Hash matching

Technical reporting is often an approach taken in the preliminary stages of cases involving indecent images of children [5] where the use of hash matching techniques can speed up the identification of illegal imagery on a suspect device [23]. When conveying hash match information, the following structure is offered:

Utilising <*tools name & version number*> all reported files present on <*exhibit device*> have been hashed and their hash values compared to those values stored in our illegal imagery hash dataset <*include a relevant description of the dataset*>. It must be noted that only illegal images that have previously been hashed (“known images”) and these values having been added to our illegal imagery hash dataset can be identified via this automated process. Unknown or first‐generation images will not be detected by this process. This process completed <describe completion status i.e. fully, partially, list of errors & impact of these on any results…>. The process reported <*X number*> of files containing hashes which were also present in our illegal imagery hash dataset. Details of these files are provided here <*details of files reporting hash matches*>. Full details of the configuration of the hash analysis process run here are available upon request.

The above example provides a statement for hashing “all reported files on a device,” however, this may be adapted to reflect how any hash process has been used within the confines of a case. The effectiveness of hash matching processes is often determined by two things, first, the quality of the backend hash‐match database used, and the configuration of the hashing process. It is suggested that the technical report should describe the backend hash‐match database used in the process, for example, if a Child Abuse Image Database (CAID) package has been used, as well as the database's metadata including the number of hashes contained and the last time it was updated. Similar to the discussions of file recovery and keyword searching processes noted above, the statement should state that full details of the configuration of the hash analysis process are available upon request. Finally, consideration of the limitations of hashing matching must be made as in some cases, hash matching processes may not be suitable, for example, where a defendant is suspected of creating imagery rather than just possessing it [5], where in such cases, hash‐matching may not give a complete overview of a case.

#### 3.1.5 “Data discovery” processes

Particularly in relation to mobile devices, practitioners may utilize tools that scan a dataset for the presence of multiple artifacts that can be associated with a number of applications/software in order to potentially discover the presence of specific content [24]. Such approaches include tools like Magnet Axiom. While these tools are configurable, they allow the practitioner to effectively conduct a “find anything/everything” styled search and parse of data causing the tool to search for all structures known to it. These processes/procedures are coined here as “data discovery” processes/procedures as the practitioner may not use the tool to target specific known data in a data set, rather use the tool to see what might be present. When reporting the use of these tools, the following structure is suggested:

<*Tools name & version number*> has been used to process data from <*exhibit reference*>. This tool contains a range of inbuilt functionality designed to identify and parse information from a range of applications/software, where <*description of functions*> have been deployed. Full details of the tool and its configuration, when used as part of this examination, can be provided upon request. This process was run and completed <*describe completion status i.e. fully*, *partially*, *list of errors & impact of these on any results..*.>. On completion, the following information was presented: <*description of presented findings*>

The range of functionality deployed in regard to a data discovery process/procedure can be vast, making it difficult for a client to ascertain the scope and depth of the examination work carried out. Where possible, the practitioner may consider providing an accessible overview of how the tool was deployed (for example, whether only a subset of functionality were deployed) within the report itself, while also having full details available on request.

When reporting upon the use of data discovery processes/procedures, it is important to convey that any findings are due to the processes/procedures, as, at this point, the practitioner may not have validated their presence in the data. This should pose a limited risk as any tool used should be validated, but one that should be acknowledged.

### 3.2 Reporting the results presented by a process/procedure (the tool used)

One of the fundamental traits of a technical report lies with the practitioner presenting the results of their work as observations and technical descriptions. Therefore, it must be clear that when any results are provided due to a process/procedure being run, the report clearly conveys that this is the case. For example, a practitioner may utilize a process/procedure to extract all Internet history from a device and present it all to their client for review. Similarly, a practitioner may provide their client with all of the deleted files that have been recovered by their chosen tool. This prevents a client from over‐relying upon the results if in reality there is scope to conduct further, more thorough examination. Practitioners should consider how any results may be appropriately provided to their client, given that in some cases this may relate to large volumes of data.

However, in some cases, a practitioner may have the necessary instructions from their client to specifically highlight data and provide a more focused set of results as part of their report rather than producing the results of any process/procedure in their totality. This task requires some exploration and be approached with caution.

### 3.3 Any data specifically highlighted by the practitioner and the criteria used to do this

The Forensic Science Regulator [4 at p.47–48] emphasizes that technical reports offer “no opinion on how the files came to be on the device or whether any of their content is relevant to a fact in issue in the case.” At this point, the task of determining the meaning and the relevance of data should be separated from the task of performing a data sift using objective criteria or instructions in order to highlight it to a client and reduce their task of reviewing content. The third core construct of a technical report concerns those situations where the practitioner is required to conduct a sift of data resulting from the technical processes/procedures they have conducted or data they have seen in a dataset. This may help to reduce the amount of data being supplied to the client, where it is important to draw a clear line as to what this task should and should not involve. Of course, in some circumstances, it may be more appropriate to supply all data generated as a result of a forensic process being run to a client, leaving them with the task of interpreting it.

While in some cases, a practitioner may conduct processes/procedures that result in packages of data being produced that are then subsequently provided to an analyst or officer who is part of the wider investigatory team for their evaluation, in other instances, an initial sifting of data may be instructed. This “sifting” should be a process driven by a set of objective criteria (“the instructions”) provided by a client or determined by a wider investigatory team so as to acknowledge data that is relevant to an inquiry. The sifting process should not involve the practitioner having to establish the meaning of any data beyond a technical level of description. Any criteria used to sift a dataset should be fully described by the practitioner in their report so that it is clear the sift was conducted.

To provide context, a practitioner may extract all Internet history from a suspect device's browser, resulting in 100,000 records requiring review. While all records could be provided by the practitioner for review by someone other than themselves, they may receive instructions from their client to only produce records showing reported “visits” to specific websites. In such cases, the practitioner may seek to cut down the number of results presented to a client by using this sifting criterion. This may be considered their objective criteria for determining relevance and conveying this information may be done in the following way:

Instructions provided by <*client details*> permitted all Internet history records discovered on <*exhibit reference*> and presented by <*tools name & version number*> to be sifted based upon <*list of criteria*>. The sifted results are as follows: <*list of sifted results*>. All Internet history records can be provided on request.

Any sift criteria must be capable of being applied without requiring the practitioner to interpret the meaning of data beyond a technical level. Therefore it is suggested that sift criteria should be applied to any file/record metadata or its content. In relation to file metadata, for example, a client may require a sift based on specific file types, file names containing specific criteria, files from specific locations on a system or files with timestamps aligning to a specific period of activity. Similarly, in relation to record metadata, for example, chat records may be sifted based upon when they are reported to have occurred or who with (i.e., reported contact number/name). In relation to specific content, a client may advise a practitioner to report any file which is found to contain specific information.

While practitioners may be required to sift data sets generated from automated processes/procedures run, they may also be required to make judgments on the need to report upon single files found during their manual scrutiny of a system. It is suggested that in such cases, similar objective criteria must be applied. Consider the following example of reporting upon an assumed DVD list file in a suspected case of DVD piracy:

The file “*The list.docx*” is located at file path “*\Users\Smith\Desktop*.” This location is where files on the “Desktop” of the User Profile “*Smith*” are stored. This file is a word processing file with a reported created date of <*created date*> and a last accessed date of <*last accessed date*>. Some word processing files contain internal information that describes what is termed the “*author*” of a document. Client instructions require all files making reference to the “production or sale” of DVDs to be reported. Following an examination of the file “*The list.docx*”, author information is contained and displays a value of <*author name*>. This file contained text with the words “DVDs for sale” and contained titles to known DVDs available for purchase in commercial shops. This text matched the provided criteria for determining relevant files and has therefore been reported. This file is available as <*exhibited file*>.

The sift processes should be as far as possible transparent and client instruction‐driven (by those forming part of a wider investigative team who have in depth knowledge of any case's circumstance), it should not be left entirely to the practitioner to determine what and what not to include in a report. This requires them to interpret the value and meaning of data in regard to the inquiry, arguably beyond the remit of the task of reporting at a technical level. For that reason, it could be argued that the technical report offers limited value to a case. While this may be true, it does reflect the amount of work undertaken, where technical reports may offer more timely information in comparison to reporting at other levels. A client must determine what they need from an investigation in order to progress, where in some cases, a technical report may be unsuitable and a practitioner must be commissioned to provide interpretive guidance.

To note—terminology: In the above example, the terms “created date” and “last accessed date” are used. To help clients to understand the contents of the report, practitioners should consider the use of supplementary glossaries that outline agreed and standardized definitions of key terms used in their reports. In this case, a supporting glossary may accompany a technical report that defines what the terms “created date” and “last accessed date” mean. This approach is recommended by SWGDE [26, 27].

## 4 DISCUSSIONS AND CONCLUSIONS

Technical reports are intended to be vehicles to convey technical and objective case information, often commissioned as part of initial case intelligence gathering processes to offer timely information [28]. Therefore, perhaps the first point to address is ensuring that for any given case, reporting at a technical level is both suitable for and the service that is required by the client. Any technical report content should be clear and accessible to its readers, reporting only upon technical meaning, absent of opinion. Often this is for the purpose of supporting any decision‐making by an investigative team with regard to how to progress with an inquiry. For this reason, technical reports should be concise and unambiguous in the language they use to communicate any findings. Technical results are also a product of the work conducted, where at an initial case intelligence stage, often findings are derived from the use of processes/procedures which are used to search, sift and highlight potentially useful data. Any interpretation of data beyond the basic reporting of technical information and description likely requires additional hypothesis testing and evaluation, tasks which are often conducted later in an investigative process should they be needed and arguably less often at the initial intelligence‐gathering stages. For that reason, technical reports must be clear when communicating findings that they are presenting them without any interpretation of meaning beyond a simple technical description.

Some may raise the question—“*but why does it matter how findings are worded*?” A response centers upon the need for quality assurance. While in some cases, loosely worded technical reports may still result in a client forming the correct judgment with regard to any findings allowing them to progress an investigation effectively, achieving this outcome should never be left to chance. Being explicitly clear removes any room for the misinterpretation and/or unintended interpretation of any findings presented from an examination by the client. This in turn helps to prevent future procedural and decision‐making errors in relation to the case. All branches of forensic science operate in order to support legal systems to provide fair and reliable justice where measures that seek to ensure this occurs, and with greater consistency should be encouraged. We must also consider that through an explicit tightening of the language and structure used to communicate findings at the technical level, and by doing this consistently, the emergence of frivolous arguments may be prevented which may be deployed in an effort to generate uncertainty as to the reliability of any findings presented, in instances where this would not have been possible if any content had been conveyed more comprehensively. Resources such as standardized and accepted glossaries of terms may support the process of clarifying language usage in reports [27].

It is by no means suggested that the current state of technical reporting in DF is bad; speculation as to the quality and consistency of this practice is largely unknown and may never be wholly evaluated due to the legal and logistical challenges for conducting such an evaluation. However, reporting practices may be divergent and with that comes inevitable poor practice. While certain jurisdictions may have their own initiatives, which help to support consistent and quality practices, field‐wide guidance of conducting reporting in DF is limited. Therefore, this work intends to start discussions in the area of technical reporting and begin to suggest what is intended to be good practices while raising potential areas of challenge. This work encourages practitioners to scrutinize their technical report content in order to identify and prevent poor reporting practices from persisting within their organizations. The use of peer‐review measures will also help to perform this assessment [16, 32]. In turn, it is recognized that self‐evaluation is a difficult thing to conduct and therefore it is hoped that the discussions offered in this work can support organizations and their practitioners to be reflective of their current practices and identify whether any areas of improvement exist. Moving forward, it is suggested that the DF would benefit from the development of overarching and agreed standards for reporting.
